# The gamma-band activity model of the near-death experience: a critique and a reinterpretation.

**DOI:** 10.12688/f1000research.151422.2

**Published:** 2024-09-17

**Authors:** Nigel A Shaw

**Affiliations:** 1 Department of Anatomy, University of Auckland, Auckland, New Zealand

**Keywords:** Amygdala, Consciousness, Electroencephalogram, Electromyogram, Gamma oscillations, Near-death Experience

## Abstract

Near-death experience (NDE) is a transcendent mental event of uncertain etiology that arises on the cusp of biological death. Since the discovery of NDE in the mid-1970s, multiple neuroscientific theories have been developed in an attempt to account for it in strictly materialistic or reductionistic terms. Therefore, in this conception, NDE is at most an extraordinary hallucination without any otherworldly, spiritual, or supernatural denotations. During the last decade or so, a number of animal and clinical studies have emerged which reported that about the time of death, there may be a surge of high frequency electroencephalogram (EEG) at a time when cortical electrical activity is otherwise at a very low ebb. This oscillatory rhythm falls within the range of the enigmatic brain wave-labelled gamma-band activity (GBA). Therefore, it has been proposed that this brief, paradoxical, and perimortem burst of the GBA may represent the neural foundation of the NDE. This study examines three separate but related questions concerning this phenomenon. The first problem pertains to the electrogenesis of standard GBA and the extent to which authentic cerebral activity has been contaminated by myogenic artifacts. The second problem involves the question of whether agents that can mimic NDE are also underlain by GBA. The third question concerns the electrogenesis of the surge in GBA itself. It has been contended that this is neither cortical nor myogenic in origin. Rather, it arises in a subcortical (amygdaloid) location but is recorded at the cortex via volume conduction, thereby mimicking standard GBA. Although this surge of GBA contains genuine electrophysiological activity and is an intriguing and provocative finding, there is little evidence to suggest that it could act as a kind of neurobiological skeleton for a phenomenon such as NDE.

## Introduction

The near-death experience (NDE) is a constellation of mental events often of a mystical or transcendental quality which uniquely arise on the precipice of biological death. A subject, victim, or patient is typically unconscious or semi-conscious. What also distinguishes NDE is its recurring nature. Once recovered, survivors tended to report remarkably similar sensations, perceptions, and usually (although not invariably) positive feelings. Although recorded for millennia in almost every society, culture, and religion, formal contemporary studies of the NDE did not begin until the mid-1970s. The catalyst was the publication of psychiatrist Raymond Moody’s book “Light After Light” ( Moody, 1975). When considering NDE, it is probably not useful to conflate it with other near-death phenomena such as the deathbed vision and the anticipated or fear of death experience, even though their features may overlap.

There is little doubt that NDE is a *bona fide* experience ( Fischer and Mitchell-Yellin, 2016; Sartori, 2016). The principal question is what the ontological status is. Sometimes, the sequence of NDE components unfolds in a manner that creates the impression of an other-worldly journey. This has been interpreted by some as the first step towards a post-mortem existence and, therefore, a glimpse of life after death ( Wheeler, 1977; Blackmore, 1993). *Prima facie,* the NDE might, therefore, provide a kind of empirical confirmation of the notion that mind, spirit, soul, or consciousness are quite separate entities with a distinct existence or at least independence from the corporeal body or brain.

Contrary to such, a survivalist or supernatural paradigm is a naturalistic or materialistic explanation that maintains that the NDE is, at most, a complex and fantastic hallucination ( Sacks, 2012). The primary difficulty with this proposal is accounting for how phantasmagoria could arise under such inauspicious circumstances. During the past half-century, at least two dozen neuroscientific theories have been proposed. There are many variations of one another.

Earlier models tended to rely on seemingly simple modes of action involving, for example, endorphins, birth memories, hypoxia, autoscopy, hypercapnia, and pharmacology. More elaborate and sophisticated theories have relied on advances in neuroscience (e.g. Saavedra-Aguilar and Gomez-Jeria, 1989; Jansen, 2001; Strassman, 2001). To date, however, no single neuroscientific theory has proved entirely satisfactory. This failure has sometimes been used as *de facto* evidence in favor of a paranormal explanation. Multiple accounts of these theories are available, and some are more sympathetic to a neuroscientific explanation than others. The selection includes Moody (1975), Ring (1980), Sabom (1982), Zaleski (1987), Morse and Perry (1992), Blackmore (1993), Fenwick and Fenwick (1995), Kellehear (1996), Ritchie and Sherrill (2007), Greyson (1998), Jansen (2001), Fox (2003), Mobbs and Watt (2011), Alexander (2012), Sacks (2012), and Michael et al. (2023). The majority of these are critical surveys of extant models, although there are some exceptions (e.g. Blackmore, 1993; Jansen, 2001; Michael et al., 2023).

In the present century, two new neuroscientific models have emerged. As a measure of popular interest in this subject, both have attracted a good deal of attention in general news media. The first is the rapid eye movement (REM) sleep-intrusion theory ( Nelson et al., 2006). More recently, the gamma oscillation model was proposed by George Mashour et al. at the University of Michigan ( Borjigin et al., 2013a). For the present purposes, this model is referred to as the gamma band activity (GBA) theory, although this was never a term formally adopted by the authors. This is also known as the end-of-life electrical surge (ELES) model ( Chawla et al., 2009). Unlike the REM-intrusion model, the GBA theory is essentially new and, in some respects, quite startling material. This was recently described as a “landmark” discovery ( Shlobin et al., 2023). Judging by a recent review, the GBA model of NDE generation seems already to have acquired a formal and respectable status whose putative merits are taken for granted ( Fritz et al., 2024).

As an example of worldwide attention the GBA model almost immediately attracted, on August 14, 2013, *The New Zealand Herald* ran a headline entitled “ *Brain kicks in as heart fails”.* The article then began “Scientists believe they may have solved the mystery behind the shining light people often talk about after having near-death experiences.”

The starting point of the investigation by Borjigin et al. was an attempt to solve a neurophysiological puzzle. A significant minority of patients who suffer cardiopulmonary arrest (CPA) and survive also report having NDE ( Parnia et al., 2001; van Lommel et al., 2001). With cerebral blood flow failing, the acute loss of O _2_ supply and depletion of glucose reserves means that cortical function, as reflected in electroencephalographic (EEG) activity, will rapidly cease. The characteristic pattern consists of a continuous slowing of frequency and loss of amplitude, which rapidly deteriorates towards a complete loss of electrical activity, that is, to an isoelectric EEG ( Clute and Levy, 1990; van Lommel 2004). However, if NDE is a genuine type of brain hallucination, it would be predicted that there should still be at least a transient neural correlate of such an event that might be detectable in a state of disconnected consciousness despite the state of cerebral ischemia. Throughout this review, amplitude refers to activity measured in microvolts.

Therefore, the authors focused their attention on an enigmatic high-frequency EEG rhythm known as gamma oscillation. This activity has long been considered to be related to the maintenance of consciousness and states of alertness ( Thompson, 1975). Borjigin et al. reasoned that a brief paroxysmal burst of gamma oscillations in the dying brain might plausibly represent what they described as the neural signature of NDE.

## Functional significance and generation of gamma waves

Hans Berger, the discoverer of human EEG, defined low-voltage fast cortical oscillations with a frequency greater than alpha as beta activity, that is, 14 Hz or greater. However, pioneering electroencephalographer Herbert Jasper soon identified an even higher frequency EEG, which was labelled as gamma rhythm ( Jasper and Andrews, 1938). The exact constituent gamma frequency is unclear, but most contemporary research studies define it as oscillatory neural activity with a frequency range of 30–100 Hz, which is normally split into low, medium, and high gamma ( Herrmann et al., 2004; Hoogenboom et al., 2006; Jensen et al., 2007; Buzsaki and Wang, 2012).

All types of higher cognitive processes have now been partially attributed to synchronized high-frequency GBA. Nonetheless, even the most partisan advocates of such a role usually acknowledge that the current understanding is still fundamentally speculative and tentative. Among the cognitive operations frequently thought to be involved with GBA are perception ( Tallon-Baudry and Bertrand, 1999; Lee et al., 2003; Kaiser and Lutzenberger, 2005; Hoogenboom et al., 2006; Uhlhass and Singer, 2006; Hughes, 2008; Ahmed and Cash, 2013; Bosman et al., 2014) selective attention ( Kaiser and Lutzenberger, 2005; Uhlhass and Singer, 2006; Jensen et al., 2007; Fries, 2009; Ahmed and Cash, 2013; Bosman et al., 2014), long and short-term memory encoding and formation ( Kaiser and Lutzenberger, 2003, 2005; Herrmann et al., 2004; Uhlhass and Singer, 2006; Jensen et al., 2007; Hughes, 2008; Bosman et al., 2014), sensory-motor control ( Lee et al., 2003; Uhlhass and Singer, 2006; Schroeder and Lakatos, 2009; Ahmed and Cash, 2013; Bosman et al., 2014) and language acquisition ( Lee et al., 2003). These are in addition to the long-standing view that GBA is involved in arousal and conscious awareness ( Lee et al., 2003; Hughes, 2008; Ahmed and Cash, 2013) Most notably, oscillatory gamma rhythms are considered to play a crucial role in sensory processing, particularly regarding the so-called perceptual binding problem ( Tallon-Baudry and Bertrand, 1999; Lee et al., 2003; Jensen et al., 2007; Hughes, 2008; Schroeder and Lakatos, 2009). This involves a mechanism by which separate features, elements, or components are successfully combined, coordinated, and processed to form a coherent and integrated percept. According to the current doctrine, this is accomplished by synchronized gamma – frequency rhythms activating separated, distributed, and diverse neuronal networks, assemblies, and regions. This facilitates successful perception or internal representation of a particular object or image. Presumably, synchronized GBA would lower the discharge threshold of the target neurons.

In addition to controlling and recruiting neuronal ensembles, gamma frequency synchronization plays a role as a timekeeper ( Buzsaki and Wang, 2012). In this sense, it provides a temporal framework for information processing and communication within the brain, which acts as a neural clock.

As with its functional role, there is still no definitive understanding of the cellular mechanisms responsible for the electrogenesis of GBA ( Traub et al., 1999; Lee et al., 2003; Traub et al., 2004; Uhlhass and Singer, 2006; Bartos et al., 2007; Fries, 2009; Traub and Whittington, 2010; Buzsaki and Wang, 2012; Ahmed and Cash, 2013). Both excitatory and inhibitory chemical neurotransmissions are likely involved in this process although gamma oscillations may also be regulated via electrotonically-coupled gap junctions. However, the omnipresence of GABAergic interneurons in networks and regions where gamma waves typically arise has encouraged the idea that these are the basic functional units of high-frequency oscillations.

The standard operating model of cortical gamma waves is usually referred to as the PING (pyramidal interneuron network gamma) system. According to this conception, gamma oscillations are the product of a dynamic relationship between fast-spiking GABAergic interneurons and pyramidal cells residing mostly in cortical layers II and III ( Brunel and Wang, 2003; Bartos et al., 2007; Buzsaki and Wang, 2012; Sanacora and Schatzberg, 2015; McMillan and Muthukumaraswamy, 2020). This process, in turn, is controlled by inhibitory and excitatory interneuron receptors. Receptor antagonism and subsequent disinhibition, for example, raises excitation levels among the pyramidal population thereby generating and driving higher amplitude trains of gamma waves. Further, continual mutual inhibition among the interneurons may set up recurrent feedback loops that autonomously maintain the periodicity, synchronicity and frequency of the gamma rhythms. The ratio of the relatively small number of interneurons to the more numerous pyramidal cells may also help to account for and maintain the synchronous patterns among GBA.

Neurophysiological models, such as those outlined above, have been developed primarily to account for cortical gamma waves. Therefore, they should provide a framework for or background to the GBA model of NDE generation.

## Evidence for the GBA model

Borjigin et al. (2013a) used an adult rat model. In advance, bilateral skull-screw electrodes were implanted over the frontal, parietal, and visual cortices. On the experimental day, EEG activity was recorded sequentially from animals while awake, while anesthetized with a standard ketamine and xylazine combination, and during a state of cardiac arrest (induced by intracardiac injection of potassium chloride). Initially, the amplitude of the EEG was preserved and then decreased. However, beginning approximately 10 s after the onset of cardiac arrest and before the onset of suppression and essentially permanent isoelectricity, there was a brief interregnum consisting of mostly tightly synchronized, relatively high-amplitude, diffusely distributed GBA with a dominant frequency of approximately 40 Hz and a range of 25-50 Hz. This paroxysmal activity persisted for up to 20 s. Both the generalized coherence and power of these oscillations were significantly enhanced on the brink of death when compared with those in animals who were awake or anesthetized.

Based on assumptions regarding the normal role of cortical gamma oscillations in mediating cognitive and perceptual function as summarized above, the authors interpreted their findings as evidence that there exists a transitory period of intensified alertness in the acutely dying brain where complex multi-faceted information processing may still occur. They conclude their paper with the provocative suggestion that this apparent discovery of high-level conscious activity on the cusp of death might provide the neurophysiological foundation for a novel naturalistic explanation of NDE.

## Critique of the GBA model

The *prima facie* weakness of the GBA theory is that it provides only the barest and most inchoate notion of how NDE might have been triggered under such conditions. Borjigin et al. made little or no attempt to advance or expand their model beyond the observation that this anomalous surge in electrical activity can be reliably recorded from the rodent brain almost immediately following cardiac arrest. Otherwise, it is not at all obvious how or why a state of comparatively high-voltage GBA generated ephemerally at the brink of biological death could ultimately sculpt a NDE. Vague statements such as the data providing “strong evidence for the potential of heightened cognitive processing in the near-death state” are not especially edifying. However, if investigators observe such an evident and unmistakable pattern of activity in the near-death state of a mammalian brain, then this is precisely what might be expected if there were indeed some neurobiological processes underlying NDE in humans. In a subsequent rebuttal, the authors expanded upon these questions ( Borjigin et al., 2013c).

Nevertheless, if the current theories regarding the focal role that GBA is expected to play in the neuronal processing of perception, sensation, and memory ultimately turn out to be correct, then these activities might at least partially evoke the complex imagery and other phenomena of the NDE. It is more difficult to account for the invariant nature of much of the qualitative content and the apparent serial ordering of individual elements of the NDE ( Michael et al., 2023). However, more recent investigations have strongly suggested that such temporal ordering is not a common feature of most NDEs ( Martial et al., 2017). The authors did, however, report a definite ordering although in only a tiny minority of their subjects. Unfortunately, any attempt to extend, develop, or expand the GBA theory would inevitably be stymied because the body of evidence concerning the role of gamma oscillations in any higher-level cognitive function is convincing in many respects but not entirely conclusive ( Buzsaki, 2006).

A second difficulty concerns the status of gamma rhythm as a measure of sentience. Borjigin et al. seem to take for granted the standard doctrine that GBA is “associated with waking consciousness” and their model is largely predicated on this relatively long-standing assumption. There is little direct evidence linking GBA to conscious experiences. Perhaps the most persuasive argument is that gamma frequency EEG is more prevalent during states of wakefulness and REM sleep than during non-REM (slow wave) sleep or general anesthesia ( Lee et al., 2003). Borjigin also appears to endorse this straightforward and intuitive view. Nevertheless, the ubiquitous presence of gamma waves means that there is a lack of clear, consistent, and exclusive associations between gamma activity and wakefulness ( Vanderwolf, 2000; Buzsaki and Wang, 2012). This apparent independence tends to undermine the potential role of GBA as a mediator or reliable index of consciousness. Thus, if there is a functional disconnect between GBA and levels of consciousness, this would mean that any apparent relationship between them would be merely accidental or fortuitous. This raises the apostatic possibility that GBA has little, if anything, to do with the maintenance and regulation of states of awareness. The extent to which this would represent a challenge to the integrity and feasibility of the GBA model for NDE generation is uncertain. However, a central tenet is that high-voltage gamma signals are an indication of a hyperaroused brain even in the context of rapidly deteriorating and irreversible cerebral function.

The authors formally analyzed the absolute and relative power of GBA for both wakefulness and ketamine-xylazine anesthesia. For absolute power, little difference could be detected between the conscious and unconscious states for any of the four GBA subbands. Regarding relative power, there was a tendency for activity to increase during the waking period, but only in the medium and higher gamma bands. Such findings do not resolve the relationship between GBA and the level of arousal. Of course, it is possible that GBA may subserve separate roles or functions depending on the state of consciousness.

Shortly after Borjigin’s article was published, Greyson et al. produced a brief but skeptical review of its relevance for understanding the origins of NDE ( Greyson et al., 2013). This elicited a forceful rebuttal by Borjigin ( Borjigin et al., 2013c). Four of the most pertinent questions are summarized below:

First, Greyson questioned how the authors could claim that there was evidence of a hyperaroused brain in the post-cardiac arrest state when total electrical energy was drastically dissipated. Borjigin’s rejoinder reiterated the dogma that gamma oscillations play a central role in regulating wakefulness and alertness. This implies that concentrating on the overall undifferentiated EEG is misleading and immaterial in assessing the level of consciousness after cardiac arrest. Instead, they insisted that the appropriate index of conscious experience under these circumstances was the power, synchrony, connectivity, and coupling of the gamma oscillations. Judging by these findings, it stands to reason that the rodent brain must have been in a state of high alertness, albeit for a relatively fleeting period post cardiac arrest.

Second, Greyson identified a complication inherent in rodent data. All rats displayed a burst of high-frequency electrical activity; however, only a minority of patients surviving cardiac arrest subsequently reported an NDE. If the surge of EEG was really responsible for the NDE, this implies that an NDE should be a near-universal event in such patients, not an occasional one. Borjigin dealt with this objection by pointing out that their animal subjects were a very homogeneous group, in contrast to patients who reported having had an NDE. Differences among the latter with regard to genetics, physiology, and clinical conditions might account for the discrepancy in the predicted rate of NDE reporting. In addition, Borjigin also raised the possibility that many cardiac arrest patients have a NDE, but the remembrance of it is forgotten by the time consciousness is regained.

Third, Greyson was also curious as to why a burst of postcardiac arrest EEG had never been detected in earlier clinical studies. It was pointed out that EEG characteristically deteriorates and rapidly disappears within approximately 20 s after cardiac arrest ( van Lommel, 2004). Greyson claimed that there is no evidence of this surprising and incongruous EEG surge that there should otherwise have been in at least some patients. In response, Borjigin et al. suggested that the different techniques for recording and more complex processing and analysis that they employed in their own studies might account for the failure to observe and quantify any such EEG surge during clinical studies. In particular, they call attention to the fact that patient EEG recordings are performed using scalp electrodes. In contrast, animal studies used epidural electrodes, which meant that EEG signals were not attenuated or vitiated via passage through the skull and scalp. Therefore, more detailed and sensitive information about cortical function could be expected to be obtained under such conditions.

Fourth, Greyson claimed that many (possibly a quarter) NDEs arose during general anesthesia. If this estimate is correct, then the question may be raised as to why there was no evidence of post -arrest surges of GBA during the previous anesthetic state. Borjigin does not explicitly address this concern.

## Clinical studies of GBA activity

Subsequently, an activity analogous to that found in rodents was also discovered in a number of clinical recordings. The detection of these high-frequency EEG bursts during the perimortem period strengthened the idea of a putative role for gamma oscillations in the induction of NDE. In fact, Mashour et al. in 2013 were not the first to suggest that a surge in EEG activity at the moment of death might be linked to NDE. Four years earlier, Chawla et al. (2009) conducted a study that anticipated much of Borjigin’s research ( Chawla et al., 2009). Instead of rodents, the subjects were seven terminally ill patients. EEG activity was measured using either a Bispectral Index Monitor (n=6) or Sedline Monitor (n=1). These produce an integrated numerical value, thereby providing only a relative gross measure of the level of consciousness or depth of anesthesia.

All patients exhibited a brief surge in electrical energy just prior to death and immediately after the loss of blood pressure. The authors described this phenomenon as a spike that typically lasted for just 30 – 80 s. It was assumed that the spike was composed of genuine EEG activity, but raw data were only available for a single case using the Sedline Monitor. A retrospective analysis of this recording revealed that the spike was composed of high-frequency EEG signals within the gamma range. Such a finding is congruent with the rodent study, and the authors also emphasized the supposed association between gamma waves and cerebral arousal. Nonetheless, it remains uncertain whether the spikes in the other six patients also contained GBA. The authors also devoted some effort to try to exclude sources other than the EEG particularly electromyographic (EMG) artefact and electrical interference.

Subsequently, Chawla and Seneff (2013) positively wrote about rodent findings, claiming that they confirmed their original human observations. In contrast, Borjigin et al. (2013b) did not seem enthusiastic about the possible relevance of these ELES (as Chawla described them) recorded from human patients to their animal research. They listed several reasons for remaining wary. These caveats include the different methodologies used to collect and analyze the EEG and the possible neurological impairment of critically ill patients.

Similar to Borjigin, the authors also speculated that the ELES could represent the psychophysiological basis of NDE. Like the rodent model, scant attention has been paid to how the EEG spike might actually induce a NDE. According to the authors, this could involve anoxic depolarization of the neuronal membrane, creating a temporary flood of gamma-frequency EEG signals. By some unspecified operation, this activity interferes with or destabilizes synaptic connections underlying memory circuits leading to the release of key components of the NDE.

To further investigate and quantify ELES, the authors analyzed the terminal EEG of a much larger group of critically ill palliative care patients (n=35) ( Chawla et al., 2017). In this case, they employed only a Sedline Monitor to provide a measure of cerebral activity. The output from this device is labelled the patient state index (PSI). The PSI has a 1 – 100 scale and, therefore, provides an objective index of the level of consciousness. A significant increase in PSI after the cessation of cardiac function was used to classify episodes in which there was a spike or surge in EEG (i.e., an ELES). None of the patients diagnosed with brain death generated an ELES. However, almost half of the remaining participants displayed a robust spike shortly after death. The authors inferred that this surge was composed of high-frequency oscillatory signals that they implied resided within the gamma band range and was compatible with rodent findings. They also argued that bursts are unlikely to reflect high-frequency myogenic activity. It was concluded that the ELES was not an unusual event under such circumstances, but no comment was made regarding its relevance to the NDE or any other near-death phenomena.

Apart from Chawla et al. (2009), there are other potentially relevant clinical studies that predate Borjigin’s animal research. For example, Auyong et al. (2010) recorded processed EEG from three terminally ill patients using a Bispectral Index Monitor. Two of these were processed for donations after cardiac death. The Monitor generates a bispectral index score (BIS) between 0 (brain death or an isoelectric EEG) and 100 (total arousal or wide awake). A score of over 90 is an indication of at least a basic level of responsiveness ( Liu et al., 1997). Therefore, this is equivalent to the PSI method employed by Chawla et al. (2017). In all three patients analyzed, it was found that the BIS roughly doubled within minutes after withdrawal of life support and remained elevated until just prior to cardiac death when it dropped precipitously. The maximum scores recorded for the patients were 95, 84, and 83, respectively. In the face of this, it could be inferred that such values should reflect a reasonably high level of consciousness in patients despite their end-of-life status.

The authors were uncertain what to make of their findings, but did suggest that a “distinct change in cortical electrical activity may have occurred.” Notably, in one patient, the raw EEG consisted of low-amplitude fast activity during the period when the BIS was paradoxically high. The authors also draw attention to the similarity of their findings to those of Chawla et al. (2009).

Another example of a clinical recording of GBA is a single case study ( Vicente et al., 2022). The subject was an 87-year old male who suffered a traumatic brain injury, underwent a craniotomy to evacuate a subdural hematoma, and subsequently developed seizures. He experienced a heart attack and died while being continuously monitored using EEG. Fortuitously, the neurophysiological events immediately surrounding death were therefore able to be captured. The authors claimed that this was an unprecedented and unique recording. By this, they mean “the first continuous EEG recording from the human brain in the transition phase to death.”

Epochs of EEG activity, each of 30 s duration, were analyzed. During the final 900 s following the last seizure, the overall EEG activity deteriorated. However, what was most striking was the huge intensification of gamma waves in the 30 s prior to cardiac arrest. This transition period immediately before cardiac arrest was otherwise characterized by bilateral suppression of cerebral rhythms. The surge of high-frequency activity did not persist in the post-cardiac arrest period but remained relatively higher than in the interictal interval some 15 min earlier. This is in marked contrast to EEG in the other frequency bands.

Vicente et al. stressed the similarity between their findings and those of rodents. However, there was one significant difference between the animal data and other human studies of ELES ( Auyong et al., 2010; Chawla et al., 2017). In all such cases, the onset of gamma waves did not begin until after cardiac arrest or withdrawal of support. By contrast, Vicente et al. are outliers in this respect. A burst of gamma activity occurred immediately before the cardiac arrest. It is assumed that the same basic pathobiological mechanisms are responsible for the surge, regardless of timing. However, the authors made no categorical comments on this discrepancy. Cardiac arrest was defined as the sudden loss of the ECG signal. There was no mention of any pre-arrest ECG abnormality.

The authors acknowledged that comparing their EEG findings of heightened power and connectivity of their elderly and critically ill patient with healthy rodents must be performed cautiously. Nonetheless, they believed that the high-frequency surge recorded in both species might be legitimately interpreted as a neural signal that high-level cerebral function had been preserved, albeit briefly. Further, this could therefore constitute evidence that memory, perception, information processing, and dreaming networks and circuits might still be able to operate, and therefore could possibly represent the neural substrate of an NDE. Indeed, their implicit enthusiasm for such a notion led them to make quite unjustifiably premature statements such as “to investigate oscillatory changes during near-death experiences”. It takes for granted that the two are temporally related, for which there is currently no direct evidence.

As with animal data, Greyson also provided a critical commentary on these human findings ( Greyson et al., 2022). First, it was pointed out that there was little gamma power in the post-cardiac arrest period. What was observed was that gamma oscillations were relatively more prominent than the even more depressed alpha, beta and delta rhythms. Second, it was argued that identifying the exact time of cardiac arrest was a somewhat arbitrary and uncertain decision. Electrocardiographic (ECG) activity was still present, even after it was assumed that the heart had stopped beating. The remaining challenge posed by Greyson is potentially the most serious. This raises the question of whether gamma oscillations are partially artifactual rather than authentic. This controversy will be discussed later in this review.

The most recent study of the gamma surge in terminally ill patients was conducted by the same group that had carried out the original rodent investigation ( Xu et al., 2023). EEG was recorded in four critically ill comatose patients before and after mechanical ventilatory support was withdrawn. In two of these cases, there was a characteristic burst in gamma power that appeared within seconds of the loss of breathing support. As the hypoxic state worsened, the high-frequency activity waxed in strength, but then waned and eventually ceased after approximately five minutes. The authors concluded that their findings provided compelling evidence for a brief period of heightened sentience in the dying human brain. Although based on a small number of subjects, it is also notable that approximately the same percentage of patients exhibited a perimortem spike as did the much larger cohort studied by Chawla et al. (2017). It is somewhat disconcerting that the authors devote barely a single sentence to a comparison with relevant animal and clinical data.

## EEG activity underlying mystical states

Computational neuroscientist Christof Koch (2023) provided a commentary on the work of Xu et al. He suspected that high-frequency high-amplitude oscillations were contaminated by muscle and/or seizure activity. However, such skepticism is really just a reiteration of the concerns that the authors themselves have proactively dealt with. In fact, Koch’s most useful contribution to this debate was his insight that if gamma oscillations underlie mystical states, such as the NDE, then similar activity should also be recorded after intoxication with a psychedelic agent that can mimic the NDE.

Information directly relevant to this observation has recently become available, although this was not mentioned by Koch. It is well known that the classical hallucinogen *N,N*-dimethyltryptamine (DMT) (aka ayahuasca) can rapidly conjure up a mystical state very similar in terms of phenomenological features to the NDE ( Michael et al., 2023, 2024). As such, it provides the foundation for Strassman’s “Spirit Molecule” model of NDE generation ( Strassman, 2001). Another formal study recently confirmed the finding that DMT can virtually simulate the entire phenomenology of the NDE ( Timmermann et al. 2018). In his original research, Strassman abandoned EEG recordings during the DMT state after only three attempts. However, Timmermann et al. (2019) successfully recorded brain activity in healthy adults during the intense hallucinatory phase of DMT intoxication. What was discovered was the dominance of slow delta and theta oscillations accompanied by a striking loss of spectral power in the faster rhythms. Such findings are, of course, quite contrary to the expectations of the GBA model. Assuming that the high-frequency burst of activity is actually associated with the induction of an NDE, it would be predicted that fast oscillations underlie the action of an agent such as DMT. This is strong implicit evidence that the surge in GBA at the moment of death is unlikely to be responsible for NDE.

Little attention was paid to gamma activity, but what there was suggests that it did not share the same drop in absolute power as the alpha and beta waves during the DMT state. This may slightly confound the matter, but it is clear that the lower frequencies are most functionally relevant. Therefore, it follows that if the post-arrest surge was related to the NDE, it should have consisted predominantly of delta and theta waves, albeit with a contribution from gamma. In a current study, where EEG and functional MRI activities were simultaneously recorded ( Timmermann et al., 2023), essentially the same findings were obtained.

This relationship between hallucinatory experience closely resembling NDE and low-frequency EEG is even more striking with dissociative anesthetics. Rogo (1989) provided multiple illustrations of how intoxication with phencyclidine (PCP) and ketamine can reproduce the imagery and phenomenology of NDE. Similarly, Jansen’s NMDA model of NDE generation relies on the assumption that a congener of ketamine is intrinsically synthesized ( Jansen, 2001). This mode of action is similar to that of endogenous DMT ( Strassman, 2001). However, unlike DMT, there are numerous examples of raw unprocessed EEG that underlie ketamine or PCP anesthesia. Without exception, they are characterized by slow diffuse activity within the theta range (e.g. Rodin et al., 1959; Corssen et al., 1974; Stockard et al., 1976).

This appears to be a general principle. In his essays on his personal experience with the classical hallucinogen mescalin, Huxley drew attention to the similar visionary properties of carbon dioxide therapy (CDT) aka carbogen ( Huxley, 2004). This treatment for mild personality disorders was introduced by the Hungarian-American psychiatrist, L.J. von Meduna, in the 1950s ( Meduna, 1950). Meduna demonstrated that after a little more than two dozen inhalations of the carbogen mixture, an ecstatic, otherworldly state almost indistinguishable from NDE could be rapidly induced in his subjects. Subsequently, Morrice (1956) recorded the EEG during the CDT and reported that it was also associated with a widespread theta rhythm.

The EEG findings of these three distinct hallucinogenic agents are therefore quite incompatible with the concept and expectation that a burst of high-frequency gamma waves might represent the psychophysiological basis of a mystical experience, such as the NDE. Nevertheless, this conclusion should be tempered with a degree of caution, as it is derived from mostly visual inspection of unprocessed EEG. With quantitative EEG analysis, there is evidence of increases in gamma band power in both humans and animals following ketamine or phencyclidine administration ( Jones et al., 2014; Shaw et al., 2015; Kao et al., 2017; McMillan and Muthukumaraswamy, 2020). There are similar results with human subjects when intoxicated with DMT ( Schenberg et al., 2015; Pallavicini et al., 2021) thereby confirming the research of Timmermann et al. discussed above. At the least, such findings are compatible with the idea that high frequency oscillations could underlie and potentially evoke psychotomimetic or psychedelic experience.

It should also be acknowledged that the notion of the advantages of a psychedelic model of NDE generation had already been exhaustively canvassed by Michael before Koch’s rather inchoate suggestion ( Michael, 2021a, 2021b). This was within the context of two commentaries on Bruce Greyson’s book *After* ( Greyson, 2021). This work is not sympathetic to psychedelic explanations or perspectives. Michael has argued, however, that psychedelic agents can not only simulate NDE but may also provide insights into their neurobiological substrate, which potentially may play a role in endogenous hallucinogens.

## Summary of clinical studies

As discussed above, the trajectory of EEG activity following cardiac arrest is both well defined and simple. It consists of an almost immediate decline in EEG power, which culminates in a state of isoelectricity within 20 s. During the last decade, however, it has become increasingly clear that in a subset of such patients, there is a sequence of fast, relatively high-amplitude activity within the frequency range of gamma oscillations superimposed on this basic pattern. Although originally described in rodents, this neurophysiological event that may arise during the perimortem period has now been reported in at least five clinical studies, as summarized in the present section. This surge, spike, or burst of activity in both rodents and humans suggests that it is probably a universal feature of the dying mammalian brain. While common in critically ill humans, it is not invariably present, probably because of the heterogeneous conditions of such patients.

Another slightly anomalous feature is that the onset of a burst or surge tends to vary among studies. This discrepancy is most likely due to the criteria used to define the moment of death ( Greyson et al., 2022). These could include undetectable blood pressure, cessation of heartbeat, loss of ECG activity, or withdrawal of life support. This is a crucial problem, as it relates to the perennial question of exactly determining when the NDE occurred.

Despite much speculation, these seemingly paradoxical surges in electrical energy about the time of death remain a phenomenon in the search for a function, origin, and significance.

## Are gamma oscillations a homogeneous type of cerebral rhythm?

The first part of this appraisal dealt with the evidence for the GBA model of NDE and some of the shortcomings, limitations, and questions raised by the theory. However, none of these matters are necessarily fatal objections nor are they inherently insoluble or irrefutable. Regrettably, a more fundamental problem also exists that could pose an existential threat to the viability of the model. This is the question of the exact origin of the gamma oscillations. In particular, whether, they are contaminated by artifacts from various sources. Recently, several of these so-called gamma skeptic papers have investigated this possibility. Three such studies are considered here.

First, Whitham et al. (2007) used neuromuscular blockade in two male subjects to quantify the contribution of EMG artifacts to higher-frequency scalp-recorded EEG. When the subjects were paralyzed with arrested skeletal muscle activity, the power of the frequencies within the gamma range decreased 10 – 200 fold. However, the precise amount depends on the particular subject, the frequencies examined, and the position of the electrodes. Nonetheless, the authors estimated that EMG activity arising from the cervical, scalp, jaw, and cranial musculature had a significant impact on the activity in the gamma frequency band. They concluded that studies of GBA recorded via scalp electrodes need to be treated carefully because of the amount of EMG activity they appear to contain.

Another study also implicated myogenic rather than neuronal activity in the generation of GBA ( Yuval-Greenberg et al., 2008). In this instance, ocular muscle activity is reflected in fast involuntary miniature eye movements or microsaccades. The normal and essential role of saccadic movements in perception is to maintain the viability of a static retinal image. In their absence, optically stabilized images rapidly fade.

Following a period of fixation, young adult subjects were presented with an image and the induced gamma-band response (iGBR) was measured. This is an augmented train of GBA occurring approximately 200-300 milliseconds post visual stimulus. iGBR is considered a crucial neural correlate of memory, perception, and other cognitive processes. Concurrent with EEG recording, binocular eye-tracking technology was employed, thereby demonstrating a strong association between the occurrence of saccadic movements and iGBR. The authors concluded that evoked GBA was little more than the far-field reflection of microsaccades. However, they were careful about extrapolating their findings beyond the scalp EEG recordings.

Despite the matter discussed above, which suggests that much of the GBA can be accounted for by non-cerebral activity, it has usually been presumed that there remains a residue or core of such activity, which reflects a genuine cerebral cortical rhythm. However, even this qualified and restricted claim has been cast into doubt. For example, Burns et al. (2011) investigated the possibility that gamma oscillations are merely filtered neural noise.

EEG recordings were obtained from several electrode positions within the primary visual cortex of curarized and anesthetized macaque monkeys, following full-field drifting grating stimulation. This consists of brief presentations of sine wave gratings moving at right angles relative to the orientation of the gratings. Bursts of post-stimulus GBA were analyzed for their degree of autocoherence. Autocoherence is defined as the constancy and regularity of the frequency and phase synchronization of the oscillations. A high level of autocoherence is a necessary property of a cerebral rhythm if it is to perform usefully and reliably as a timekeeper and therefore to subserve higher cognitive functions such as those currently attributed to GBA.

The visual cortex recordings were compared to two sets of manufactured data labelled as the noise model and the burst model. The noise model consisted of bursts of gamma activity, independent of any visual stimulation. The burst model had epochs of autocoherent gamma (or time signals) embedded in an otherwise spontaneous non-evoked activity. Burst frequency-burst duration distributions were computed for each of the three conditions. The distribution of actual cortical recordings was nearly identical to that of the simulated noise model. However, both visually and statistically, the distribution of the actual data appeared to be markedly different from that of the simulated burst model. The authors concluded that the autocoherence in GBA recorded from the visual cortex was no greater than chance and therefore, no genuine clock signals could be detected in gamma oscillations. This means that the popular concept of gamma oscillations as a type of clock-like temporal regulator and therefore synchronizing agent is unlikely to be correct and should be abandoned. Intrinsic network filters operating on neural noise were presumably responsible for recruiting and generating a prevailing gamma rhythm of approximately 40 Hz.

The authors were specifically concerned with the relevance of their findings to the binding problem and the related cognitive and perceptual functions. However, there is a broader and possibly more important message in the data. It is feasible that even so-called *bona fide* gamma oscillations arising unequivocally within the CNS may be nothing more significant or meaningful than the fortuitously created by-product of spontaneous firing and other random neuronal processes. In a subsequent experiment, Burns et al. replicated their findings in awake and anesthetized monkeys ( Xing et al., 2012).

In summary, these three studies represent an extreme position where gamma oscillations are essentially or mostly artifactual and non-functional biorhythms masquerading as authentic EEG signals. Although not mainstream, such studies are nonetheless useful in reiterating that gamma activity is not a pure cerebral rhythm. A much less controversial and nuanced perspective is that GBA is best conceived as an amalgam of genuine EEG plus EMG artifacts and/or eye movements and/or routine neural litter accidentally organized into an approximate 40 Hz pattern. The relative contribution of each to a specific or individual gamma oscillation probably varies depending on the recording conditions, nature of the subject, and their behavioral state. If this analysis is correct, then a mysterious burst of GBA occurring at the time of death must be treated cautiously and interpreted carefully. The more the GBA has been contaminated by artifacts, the less likely it is to fulfill its purported role as a kind of neural blueprint for NDE. It is a concern that those who propose a link between GBA and NDE choose to turn a proverbial blind eye to such a potential flaw or weakness in their argument.

Additional information on the likely contamination of the bursts of GBA by EMG activity was discussed by Greyson et al. (2022). A review of the difficulty in distinguishing EMG activity from high-frequency EEG is available in Muthukumaraswamy (2013).

## The canine model of alpha coma pattern

Irrespective of whether gamma activity is of myogenic or neurogenic origin, it seems very unlikely that the surge in GBA in the perimortem period reflects muscle activity. The reasons for this lack of EMG contamination have been reported previously ( Chawla et al., 2017; Xu et al., 2023). The evidence for this includes studies where animals were killed by decapitation, analysis of high PSI scores in patients in whom an ELES was observed, and complete absence of movement or EMG artifacts in unconscious subjects. This implies that the GBA surge must represent genuine brain activity; however, when it is also considered that the EEG is at such a low ebb, it suggests that the surges are composed of neither cortical EEG nor EMG activity, but rather represent activity from a third source. The question might, therefore, be asked about their actual origin. Thus, there may be a suitable candidate.

Despite the claim to the contrary, Borjigin’s rodent study is not unique. Three decades ago, a group of Russian physiologists performed an experiment that, in important ways, anticipated much of the later investigation ( Gurvitch et al., 1984) although with two chief methodological differences. First, the Russian study used canines, rather than rodents. Second, the dogs were temporarily revived after circulatory arrest. Notwithstanding these differences, the findings are consistent. It should also be noted that the present explanation of Gurvitch’s research findings differs somewhat from that in their paper. It is uncertain whether such an experiment could be performed today. However, the authors affirmed that the animals were either anesthetized or comatose throughout all procedures.

The primary aim of this study was to develop a canine model of alpha coma pattern (ACP) ( Vignaendra et al., 1974; Chokroverty, 1975; Westmoreland et al., 1975; Alving et al., 1979; Austin et al., 1988). In particular, the identification of a subcortical pacemaker could be responsible for generating such a puzzling EEG rhythm during post-anoxic coma. Clinical coma is normally associated with a standard EEG pattern consisting of predominantly generalized semi-regular high-voltage delta activity. In contrast, ACP is a comparatively rare EEG coma pattern consisting of diffuse sinusoidal rhythms, usually within the alpha or theta frequency bands, and is often a bad prognostic sign. Since alpha activity is normally correlated with a state of relaxed wakefulness, ACP may paradoxically give an erroneous impression that comatose patients may be conscious. It should be noted that while ACP helped inspire the current re-interpretation of the GBA model, it is not claimed that it plays any role in the induction of NDE.

The dogs were anesthetized with ether and had electrodes implanted at various subcortical and cortical locations. CPA was induced by ventricular fibrillation and aggressive resuscitation (cardiac massage and artificial lung ventilation), followed at various intervals. During the post-anoxic coma period, characterized by a near isoelectric EEG, bursts of high-voltage activity began to arise in the amygdaloid nuclei. This activity typically commenced approximately 30 min after resuscitation began, and individual bursts could last for several seconds. Amygdaloid discharges were reflected in closely time-locked, widely generalized oscillations recorded from the cortex. However, the amplitude of the cortical rhythms was only a fraction of that generated in the amygdala. The authors analyzed the activity recorded from other subcortical structures and established that the oscillations originated in the amygdala and were then presumed to be sequentially propagated to and distributed throughout the cortex via diffuse thalamocortical pathways. As the EEG was always within the alpha frequency band, it was also supposed that this activity could be the canine analog of ACP and, therefore, could be a prototype for the generation of ACP in human patients. In addition, Gurvitch demonstrated that if the amygdala was unilaterally or bilaterally destroyed, the alpha-like oscillatory potentials either attenuated or disappeared completely from all EEG monitoring sites. This confirmed that the amygdala was the primary site for the generation of alpha-frequency oscillations. Destruction of the amygdala was carried out by injections of Novocaine or ablation, and the location of the damage was confirmed histologically.

Whether abnormal discharges from amygdaloid pacemakers constitute the pathophysiological basis of ACP remains speculative and problematic. Also contentious is the question of how oscillatory activity is transmitted to the cortex. Considering their diminutive amplitudes, it seems most likely that the apparent alpha-range cerebral rhythms reflect far-field amygdaloid activity, which is conveyed to the surface of the brain via volume conduction. Such an origin would be much more compatible with their small size than if the amygdaloid signals had been propagated by afferent impulses through thalamocortical axonal tracts and relay stations. With the latter type of conduction and secondary generation, it might have been expected that the original high voltage of amygdaloid emissions would still have been preserved and therefore replicated at the cortical level.

If cortical oscillations represent only remotely recorded amygdaloid discharges, then one reason why they can be successfully observed during the post-resuscitation period is a lack of competition from the usually dominant cortical EEG rhythms. During cerebral ischemia, normal cortical rhythms are at least transiently suppressed, although neuronal tissue can remain reactive. However, once the cortical EEG starts to re-establish and re-assert itself, minute far-field emissions would become swamped or masked. In Gurvitch’s recordings during the post-resuscitation period, oscillatory bursts eventually began to wane, and the EEG became increasingly dominated by high-amplitude delta rhythms.

## The relevance of the canine model of alpha coma pattern to the electrogenesis of the GBA surge

Whether the canine model proposed by Gurvitch et al. can function as a viable analog of ACP in human patients remains controversial. Be that as it may, it is also possible that their findings do provide an insight into the equally baffling nature of the post-cardiac arrest burst of GBA.

Based on their paroxysmal behavior, small amplitude, and widespread cortical location, it seems reasonable to assume that the eruptions of oscillatory EEG activity recorded from both the rodent and canine cortices share a common mode and site of generation. As discussed above, the source of alpha frequency band activity was localized to the amygdaloid nuclei. Therefore, it seems logical that the bursts of GBA recorded after cardiac arrest in rodents have the same origin. Furthermore, in both instances, it is the context of cerebral ischemia or hypoxia, which allows this stream of volume-conducted amygdaloid activity to temporarily appear in cortical recordings. In addition, if far-field oscillatory activity is diffusely broadcast from the amygdaloid nuclei, this means that essentially the same signal should be received and recorded from all cortical locations. This largely accounts for the significant degree of coherence, connectivity, and synchronicity reported in both experiments.

If the preceding interpretation is correct, Borjigin’s investigation may have mistaken far-field subcortical amygdaloid activity for cortical gamma oscillations. Therefore, any comparison these investigators made between the relatively high-amplitude post-cardiac arrest EEG surge and the genuine GBA recorded during states of wakefulness and anesthesia would be quite spurious and misleading. This would also mean that no matter what the electrogenesis of gamma activity ultimately turns out to be, this would remain an irrelevant consideration. The apparent discrepancy between alpha-frequency activity generated in the amygdala and gamma-frequency activity recorded at the cortex (conflated with or in addition to cortical GBA) is discussed and potentially reconciled in a later section.

In summary, it seems possible that Borjigin and co-workers dealt with two separate rhythms that superficially resembled gamma oscillations. One is a veritable gamma rhythm with a characteristically small amplitude. In contrast, the second comprised amygdaloid activity disguised as abnormally high-amplitude fake gamma waves. The failure to recognize this flaw may have compromised their research. In particular, the increased power of the post-cardiac arrest burst of electrical energy cannot be considered a sign of heightened consciousness or alertness. Thus, if it turns out that the EEG burst is nothing more than fake cortical activity, then the question of the precise origins of gamma oscillations becomes largely redundant. This also makes it even more unlikely that GBA could serve as the pathophysiological basis of NDE.

## The origin and nature of the amygdaloid oscillations

Paroxysmal amygdaloid discharges, identified as the origin of alpha band cortical waves, have been investigated for decades ( Eidelberg and Woodbury, 1972). They have been reported in humans ( Lesse et al., 1955), chimpanzees ( Delgado et al., 1970), monkeys ( Domino and Ueki, 1960), dogs ( Domino and Ueki 1959, 1960), rabbits ( Bressler and Freeman, 1980), cats ( Pagano and Gault, 1964; Kato et al., 1964, Gault and Coustan, 1965; Knapp and Lubar, 1976, Bressler and Freeman, 1980, Bauer et al., 2007, Popescu et al., 2009), rats ( Kanta et al., 2019) and mice ( Stujenske et al., 2014). Therefore, it can be assumed that they are common properties of the mammalian limbic system.

These intermittent oscillatory emissions or signals arise in the basolateral amygdala (BLA) and consist of spindle-like bursts of high-frequency high-amplitude activity, typically lasting from seconds to minutes. They are endogenous but not strictly spontaneous because they can be evoked by a variety of stimuli. These included states of arousal, threat, learning, emotion, fear, stress, anticipation, pain, and noise. It might be predicted therefore, conditions such as hypoxia, ischemia, or cardiac arrest would provide an optimal milieu for the enhancement and/or appearance of the BLA rhythm. Nonetheless, it is in no way implied that these amygdaloid oscillations recorded from the cortex possess any mystical or psychedelic properties and, therefore, could be responsible for the NDE.

This fast BLA activity also involves a number of monickers. Sometimes, it has been labelled the 40 Hz amygdaloid rhythm after its focal frequency, or else fast oscillations of the amygdala or 40 Hz burst phenomena in the amygdala. It has also been described as amygdaloid spindling because of its waxing and waning appearance. Because its frequency normally falls within the gamma range, it has also been defined as an amygdaloid gamma oscillation.

## The effects of ether on the amygdaloid discharges

The Achilles heel of the present reinterpretation of Borjigin’s data must lie in the discrepancy in the frequency between the post-cardiac arrest bursts of electrical activity. In the Russian study, this value was mostly within the alpha band. In contrast, the American study had a dominant frequency of approximately 40 Hz, which is representative of gamma-frequency oscillations. On the face of it, this comparison exposes a difficulty which is at variance with the amygdaloid generator model proposed above. Nonetheless, this conundrum may be satisfactorily solved by one or more of the three possible factors. An interaction between these and other variables might account for the incongruity in frequencies; therefore, it is not necessarily an intractable problem.

First, the animals were subjected to quite contrary treatments, with the dogs being aggressively resuscitated. The second limitation is the use of different animal species. The 40 Hz central rhythm is not fixed, but is rather an average. Individual species may exhibit considerable variability ( Knapp and Lubar, 1976; Eidelberg and Woodbury 1972). This may range from a high frequency of approximately 50 Hz (e.g., rabbits) to a low frequency of approximately 30 Hz (e.g., humans, chimpanzees, and dogs).

The third factor is the anesthetic used. This was also a preference favored by Gurvitch. Limited information exists on the effects of ether on amygdaloid function ( Kato et al., 1964; Domino and Ueki, 1959; Gurvitch, 1964). However, it has been suggested that the amygdala is ultrasensitive to this gas. This was manifested most clearly by a reduction in the frequency of the intrinsic (40 Hz) rhythm. However, these findings are sometimes difficult to quantify. Nonetheless, overall, they indicate that even a moderate level of ether anesthesia is sufficient to reduce the frequency to within the alpha range. This would also have been a reasonable expectation, assuming that the endogenous canine rhythm is at the lower end of the mammalian scale, as discussed above. It may also be relevant that the effects of ether on rhinencephalic (although not specifically amygdaloid) function may linger for long after active inhalation has ceased ( Gault and Coustan, 1965). It can therefore be confidently asserted that the bursts of slower canine alpha activity in the cortical EEG were fundamentally and functionally equivalent to the faster bursts of the so-called gamma activity in the rodent EEG. There seems to be no essential difference once the anesthetic influence, in particular, is taken into account.

## Conclusions

The purpose of the present review was to investigate the claim that a surge in fast EEG activity during the perimortem period could serve as a neurobiological substrate for NDE. Establishing such a relationship is fraught with methodological and conceptual difficulties. Nevertheless, this paradoxical and abnormal rhythm has been detected in humans, dogs, and rats. Therefore, it can be tentatively assumed as a universal feature of the dying mammalian brain. Furthermore, it is well established that this burst of activity has an electrophysiological origin. This is not merely an artifact. However, the question persists as to not only its significance, but more fundamentally, what its electrogenesis is. If it cannot be established that it is a type of high-frequency EEG, then it is difficult to justify or understand how it could conceivably spawn an NDE.

A very fast EEG with diminutive amplitude has conventionally been labelled as the gamma rhythm. However, the present analysis has revealed that, in principle, there are multiple waveforms that superficially share most of the gamma wave characteristics. Yet, despite their common appearance, they possess distinct electrogenesis and therefore significance. One possible subtype of gamma oscillations is cortico-genic, consisting of genuine EEG activity. The second type could be of largely myogenic origin and composed of far-field muscle activity. Still, a third type could be generated by volume-conducted amygdaloid discharges. Superficially, it could be difficult to distinguish between these three near-identical potential variations or subtypes of GBA. Recognizing that the gamma rhythm may best be conceived as a generic waveform may be key to understanding the nature and origin of the high-frequency surge at the time of death.

If amygdaloid signals really are the source of the perimortem cortical paroxysms, the problem of how the transient bursts of their high-frequency activity could actually generate a NDE becomes superfluous. They could not conceivably cope with the often complex and multifarious nature of NDE with its otherworldly sights, sounds, and emotions, and dependence upon an altered state of consciousness. There seems to be little point to gain by pursuing such an unrewarding explanation.

The question of whether cortical gamma bursts reflect far-field amygdaloid activity could be definitively answered by systematic destruction of the amygdaloid nuclei in a manner similar to that employed in Gurvitch’s experiment. The preservation of the transient electrical surges under such conditions would unequivocally discredit this explanation. Nevertheless, even if an origin in the amygdala is ruled out, this would do little to improve the chances that a fleeting eruption of the GBA could underlie the NDE. This is because the genesis and relevance of the actual gamma cortical oscillations remain uncertain and disputed. It is therefore difficult to disagree with Greyson’s prescient initial verdict that the mysterious EEG burst after cardiac arrest “is unlikely to contribute to an understanding of near-death experiences” ( Greyson et al., 2013).

Nevertheless, any consideration as to whether the mysterious gamma oscillations at about the time of death are of myogenic, cortical, or amygdaloid origin may be a futile or unnecessary exercise. This is because multiple investigations have revealed that the EEG activity underlying visionary experience near- identical to the NDE lies at the opposite end of the EEG frequency spectrum to the fast gamma waves. Regardless of what the electrogenesis of the gamma spikes ultimately turns out to be, it is highly unlikely that they could be responsible for generating an NDE.

The present re-interpretation of the significance of the surges in GBA is obviously somewhat routine and quotidian, especially when compared with the more exotic, intriguing, and tantalizing alternative. It is unlikely to attract the same amount of attention from media. Nonetheless, it has the virtue of being parsimonious. As Ockham’s principle reminds us, simplicity is often a useful guide for scientific truth.

## Ethics and consent

Ethical approval and consent were not required.

## Data Availability

No data are associated with this article.
