# Supplementary material for: The gamma-band activity model of the near-death experience: a critique and a reinterpretation
Source: F1000Res. 2024 Sep 17;13:674. Originally published 2024 Jun 21. [Version 2] doi: 10.12688/f1000research.151422.2 (PMC11375408; doi:10.12688/f1000research.151422.2)
Supplement: Peer review history

# Reviewer report: Reviewer response for version 2

**DOI:** 10.5256/f1000research.171542.r324294

**Published:** 2024-09-18

**Authors:** Pascal Michael

**Affiliations:** 1 University of Greenwich, London, England, UK; 2 The Alef Trust, England, UK

I am satisfied that the author has sufficiently addressed the various comments I have most recently provided.

Is the topic of the opinion article discussed accurately in the context of the current literature?

Partly

Are arguments sufficiently supported by evidence from the published literature?

Yes

Are all factual statements correct and adequately supported by citations?

Partly

Are the conclusions drawn balanced and justified on the basis of the presented arguments?

Yes

Reviewer Expertise:

BSc and MSc in neuroscience and psychology. PhD in near-death experiences and psychedelics. Lectureship focussing on the same area of research.

I confirm that I have read this submission and believe that I have an appropriate level of expertise to confirm that it is of an acceptable scientific standard.

# Reviewer report: Reviewer response for version 1

**DOI:** 10.5256/f1000research.166067.r316189

**Published:** 2024-09-04

**Authors:** Duan Li

**Affiliations:** 1 University of Michigan Medical School, Ann Arbor, USA

This paper provides a great review of recent EEG studies on dying animals and patients and proposed alternative interpretations of gamma brain activity that was associated with near-death experiences in those studies. The article is well-written, with its conclusions supported by evidence from the referenced publications. However, I would like to suggest a few areas for improvement to further enhance the discussion and scope of the review:

1. As noted by other reviewers, several studies have reported enhanced gamma activity following the administration of hallucinogenic drugs. These findings imply that the EEG signatures of hallucinogenic agents are not entirely incompatible with the gamma surge hypothesis of NDE. The author might consider exploring this connection.
2. In addition to the work by Borjigin et al 2013, other studies have also found synchronized gamma activity in diverse rodent models, e.g. asphyxia (Li et al. 2015; Lee et al 2017, Schramm et al 2020; Carton-Leclercq et al 2023) and drug intoxication (Zhang et al 2019, in rat hippocampus). A recent review on this topic by Mashour et al. (2024) may also provide additional context.
3. Compared to cardiac arrest model in Borjigin et al. 2013, the models of circulatory arrest, especially the asphyxia and resuscitation model in Schramm et al 2020, may offer more relevant comparisons to the canine ACP model.
4. The author might consider including a discussion on potential neurophysiologic and neurochemical mechanisms underlying the gamma surge. For example, Schramm et al 2020 have investigated the neuronal origins of the EEG changes during the dying-to-recovery process. Li et al 2015 reported significant increases in the cortical release of several neurotransmitters following asphyxia.
5. Schramm et al 2020 proposed that NDE might be generated during resuscitation based on the resemblance of EEG patterns observed in hallucinatory diseases such as schizophrenia. Including a discussion on this alternative hypothesis could enrich the paper's exploration of NDE mechanisms.

Is the topic of the opinion article discussed accurately in the context of the current literature?

Partly

Are arguments sufficiently supported by evidence from the published literature?

Partly

Are all factual statements correct and adequately supported by citations?

Partly

Are the conclusions drawn balanced and justified on the basis of the presented arguments?

Yes

Reviewer Expertise:

computational neuroscience, EEG, consciousness, anesthesia

I confirm that I have read this submission and believe that I have an appropriate level of expertise to confirm that it is of an acceptable scientific standard, however I have significant reservations, as outlined above.

## References

[1] : Asphyxia-activated corticocardiac signaling accelerates onset of cardiac arrest. *Proc Natl Acad Sci U S A* .2015;112(16) : 10.1073/pnas.1423936112 E2073-82 10.1073/pnas.1423936112 25848007 PMC4413312

[2] : Neural Correlates of Consciousness at Near-Electrocerebral Silence in an Asphyxial Cardiac Arrest Model. *Brain Connect* .2017;7(3) : 10.1089/brain.2016.0471 172-181 10.1089/brain.2016.0471 28398813 PMC5399737

[3] : Near-Death High-Frequency Hyper-Synchronization in the Rat Hippocampus. *Front Neurosci* .2019;13: 10.3389/fnins.2019.00800 800 10.3389/fnins.2019.00800 31417353 PMC6684736

[4] : Identifying neuronal correlates of dying and resuscitation in a model of reversible brain anoxia. *Prog Neurobiol* .2020;185: 10.1016/j.pneurobio.2019.101733 101733 10.1016/j.pneurobio.2019.101733 31836416

[5] : Laminar organization of neocortical activities during systemic anoxia. *Neurobiol Dis* .2023;188: 10.1016/j.nbd.2023.106345 106345 10.1016/j.nbd.2023.106345 37926170

[6] : Consciousness and the Dying Brain. *Anesthesiology* .2024;140(6) : 10.1097/ALN.0000000000004970 1221-1231 10.1097/ALN.0000000000004970 38603803 PMC11096058

# Reviewer report: Reviewer response for version 1

**DOI:** 10.5256/f1000research.166067.r295790

**Published:** 2024-08-26

**Authors:** Pascal Michael

**Affiliations:** 1 University of Greenwich, London, England, UK; 2 The Alef Trust, England, UK

This is a very interesting article on reframing prior findings of gamma activity in the dying brain, with implications for the NDE, mainly by deconstructing these prior studies' interpretations and reviewing the evidence of gamma's contribution to consciousness. It is also written extremely clearly and coherently. I reviewed a previous version of this manuscript, many points of which the author has evidently incorporated well into this new one. However, there are a number of - mostly minor - strong recommendations I would finally integrate, listed below. I trust these will also be duly reflected somehow in the final version, and thus would endorse its publication.

 "confirmation of the dualist notion that mind, spirit, soul, or consciousness are quite separate entities" - I don't think that 'dualist' is justified as the only non-materialist ontology that NDEs suggest. We don't know what the specific ontological model would be, where dualism is just one (e.g. idealism, neutral monism etc)

 "an event that might be detectable in the unconscious brain despite the state of cerebral ischemia." - I wouldn't say 'unconscious', as the point of this article is that there may be such transient activity, which could be linked to the NDE event. And so, NDErs become conscious, only unconscious to external observers. Thus, 'in a state of disconnected consciousness' would be the more accurate term

 "Fast spiking inhibitory interneurons may interact and interconnect with excitatory pyramidal cells to produce and drive the GBA." - Clarify the sentence to explain how inhibitory interneuron input to the excitatory pyramidal neurons produces the GBA, if youre implying an inhibition* of the excitatory neurons

 Also this whole paragraph requires more substantiation by citations

 "It is more difficult to account for the invariant nature of much of the content " - Id put 'qualitative* content' here, and even better, 'distinguished from the phenomenological features', to drive home that such neural correlates can account for the feature invariance, but not so easily the content invariance

 "However, more recent investigations have strongly suggested that such temporal ordering is not a common feature of most NDEs ( Martial et al., 2017)" In fact, this same paper does show a definite ordering in a minority* of NDEs, approximately encompassed by sense of peace, OBE and a sense of returning

 "A second difficulty concerns the status of gamma rhythm as a measure of CNS arousal." - This whole paragraph should consider carefully if there are any conflations between 'arousal / alertness' and 'consciousness' (Seems borjigin is emphasising gamma effects on the latter)

 "The authors also devoted some effort to trying to exclude sources other than the EEG" - 'effort to try to'*

 "The Monitor generates a bispectral index score (BIS) between 0 (brain death) and 100 (total consciousness)." - Clarify if 100 ie 'total consciousness' is the average expected level for any person in a normal waking state, as this is relevant for the ELEs findings

 "By contrast, Vicente et al. are outliers in this respect. A burst of gamma activity occurred immediately before the cardiac arrest" - Clarify that you're being consistent in terms of the difference between cardiac arrest, and the onset of heart attack ie including pre-arrest fibrillation - which would weigh on this potential discrepancy 

 "The most recent study of the gamma surge in terminally ill patients was conducted by the same group that had carried out the original rodent investigation ( Xu et al., 2023)." - It would be nice to have some more sentences directly comparing findings to the previous studies, as you did more completely with said previous studies

 " *N,N*-dimethyltryptamine (DMT) (aka ayahuasca) can rapidly conjure up a mystical state virtually identical to the NDE." - This is definitely an over-statement. 'very similar in terms of phenomenological features' would be more appropriate (see Michael et al, 2023 [already cited] & Michael et al, 2024: "Smokable "Vine of the Dead": Two Case Studies of Experiencers of Both " by Pascal Michael, David Luke et al. (ciis.edu) )

 "What was discovered was The dominance of slow delta and theta oscillations was accompanied by a striking loss of spectral power in the faster rhythms. Such findings are, of course, quite contrary to the expectations of the GBA model" --> In fact, increased low gamma was found over the posterior cingulate after psilocybin administration [also shown to be very NDE resembling, see (PDF) The Death Trip: A Case Study of Psilocybin's Simulation of the Phenomenology and Long-term changes of the Near-death Experience (researchgate.net)] (Kometer et al, 2015)

 And increased low/high gamma was found in the Frontal, temporal, parietal-occipital regions, under Ayahuasca (Schenberg et al, 2015)

 Also, increased power & synchrony of gamma was found under DMT specifically, which correlated with mystical experience (Pallivicini et al, 2020)

 "Without exception, they are characterized by slow diffuse activity within the theta range (e.g. Rodin et al., 1959; Corssen et al., 1974; Stockard et al., 1976)." - In fact, the literature suggests that, although mixed due to other factors, psychoactive ketamine doses generally lead to increases in gamma --> "Ketamine amplifies induced gamma frequency oscillations in the human cerebral cortex" by Shaw et al. (2015)

 "Ketamine can produce oscillatory dynamics by engaging mechanisms dependent on the kinetics of NMDA receptors" by Nicolás-Alonso and Andrade-Valenc (2024)

 "The neurophysiology of ketamine: an integrative review" by McMillan and Muthukumaraswamy (2020)

 "This activity typically commenced approximately 30 min after resuscitation began, " - Clarify why this canine alpha pattern is relevant, given that this statement suggests it didnt even occur during (anaesthetic) coma

 "the second comprised amygdaloid activity disguised as abnormally high-amplitude fake gamma waves" - You may clarify this later, but make clear why amygdala-derived alpha diffusing to the cortex may appear as gamma (conflated with, or in addition to cortical GBA)

 " It might be predicted Therefore, conditions such as hypoxia, ischemia, or cardiac arrest... etc" + "THe effects of ether" - correct phrasing

 "Because its frequency normally falls within the gamma range, it has also been defined as an amygdaloid gamma oscillation." - Clarify why this fast BLA may simultaneously be in the gamma range but also be the origin of alpha

 "There seems to be no essential difference once the anesthetic influence, in particular, is taken into account." - May this also weigh in on Borjigin's findings that there was no GBA in the anesthetised rodents (ie anaethesia-induced significant slowing to point of gamma not being detected)?

Is the topic of the opinion article discussed accurately in the context of the current literature?

Partly

Are arguments sufficiently supported by evidence from the published literature?

Yes

Are all factual statements correct and adequately supported by citations?

Partly

Are the conclusions drawn balanced and justified on the basis of the presented arguments?

Yes

Reviewer Expertise:

BSc and MSc in neuroscience and psychology. PhD in near-death experiences and psychedelics.

I confirm that I have read this submission and believe that I have an appropriate level of expertise to confirm that it is of an acceptable scientific standard, however I have significant reservations, as outlined above.

## References

[1] : Smokable "Vine of the Dead": Two Case Studies of Experiencers of Both Changa and Near-Death Experiences. *International Journal of Transpersonal Studies Advance Publication Archive* .2024;

[2] : The Death Trip: A Case Study of Psilocybin's Simulation of the Phenomenology and Long-term changes of the Near-death Experience. *ResearchGate* .2022;

[3] : Acute Biphasic Effects of Ayahuasca. *PLoS One* .2015;10(9) : 10.1371/journal.pone.0137202 e0137202 10.1371/journal.pone.0137202 26421727 PMC4589238

[4] : Neural and subjective effects of inhaled N,N-dimethyltryptamine in natural settings. *J Psychopharmacol* .2021;35(4) : 10.1177/0269881120981384 406-420 10.1177/0269881120981384 33567945

# Reviewer report: Reviewer response for version 1

**DOI:** 10.5256/f1000research.166067.r300181

**Published:** 2024-07-29

**Authors:** Matthew R. Hudson

**Affiliations:** 1 Monash University, Melbourne,, Victoria, Australia

An interesting and comprehensive review which aimed to critically investigate evidence/suggestions that GBA is involved in near death experience, as well as provide alternative explanations for this phenomenon. In its current state, my opinion is that this article is acceptable for indexing. I do however have one comment below which the author might like to consider:

 The author describes gamma oscillatory changes following administration of hallucinogenic drugs such as ketamine and PCP and describes that these are characterised by "slow diffuse activity within the theta range", however, for both these compounds, pronounced augmentation of gamma band power has been demonstrated. Therefore, this could be considered to be compatible with the suggestion high-frequency gamma might be linked to mystical experience/NDE.

 Kao HY, Dvořák D, Park E, Kenney J, Kelemen E, Fenton AA. Phencyclidine Discoordinates Hippocampal Network Activity But Not Place Fields. J Neurosci. 2017 Dec 6;37(49):12031-12049. doi: 10.1523/JNEUROSCI.0630-17.2017. Epub 2017 Nov 8. PMID: 29118102; PMCID: PMC5719978.

 Jones, Nigel C., et al. "Effects of aberrant gamma frequency oscillations on prepulse inhibition." *International Journal of Neuropsychopharmacology* 17.10 (2014): 1671-1681.

Is the topic of the opinion article discussed accurately in the context of the current literature?

Yes

Are arguments sufficiently supported by evidence from the published literature?

Yes

Are all factual statements correct and adequately supported by citations?

Yes

Are the conclusions drawn balanced and justified on the basis of the presented arguments?

Yes

Reviewer Expertise:

Electrophysiology, neuronal oscillations, neuropsychiatry

I confirm that I have read this submission and believe that I have an appropriate level of expertise to confirm that it is of an acceptable scientific standard.

## References

[1] : Phencyclidine Discoordinates Hippocampal Network Activity But Not Place Fields. *J Neurosci* .2017;37(49) : 10.1523/JNEUROSCI.0630-17.2017 12031-12049 10.1523/JNEUROSCI.0630-17.2017 29118102 PMC5719978

[2] : Effects of aberrant gamma frequency oscillations on prepulse inhibition. *Int J Neuropsychopharmacol* .2014;17(10) : 10.1017/S1461145714000492 1671-81 10.1017/S1461145714000492 24832766
